# An Improved YOLOv8 Detection Algorithm Based on Screen Printing Defect Images

**DOI:** 10.3390/s26051604

**Published:** 2026-03-04

**Authors:** Shuqin Wu, Xinru Dong, Qiang Da, Meiou Wang, Yuxuan Sun, Ge Ge, Jinge Ma, Jiajie Kang, Yu Yao, Shubo Shi

**Affiliations:** 1 Beijing Key Laboratory of Digital Printing Equipment, School of Mechanical and Electrical Engineering, Beijing Institute of Graphic Communication, Beijing 102600, China15952171021@163.com (Y.S.); becautions@163.com (G.G.);; 2 National International Joint Research Center of Deep Geodrilling Equipment, China University of Geosciences (Beijing), Beijing 100083, China

**Keywords:** YOLOv8, screen printing defect, detection, deep learning, machine vision

## Abstract

Micro-defects, such as ink spots, scratches, and sintering formed during the screen printing process of photovoltaic cells, significantly impair module performance. Traditional machine vision methods exhibit limited detection efficiency and high false-positive and missed-detection rates, while existing deep learning algorithms struggle to achieve accurate and adaptive detection of small-target defects and background similar defects in complex industrial environments. This study proposes an enhanced defect detection methodology based on an improved YOLOv8 algorithm. A multi-focus image acquisition platform using primary and auxiliary CCDs was independently developed, integrating a high-frame-rate industrial camera and a high-resolution electron microscope, with an LED ring light employed to suppress reflections, thereby establishing a high-quality dataset covering three defect categories. The algorithm was optimized through multiple dimensions: the RepNCSPELAN4 module was incorporated into the backbone network to improve multi-scale feature fusion, and a novel wavelet transform-based WaveConv module was designed to replace traditional downsampling, thereby better preserving defect edges and texture details. The neck network integrates a lightweight shuffle attention mechanism and a new detail enhancement module to strengthen critical features while controlling model complexity. Additionally, a dedicated auxiliary detection head was added for spotting tiny ink dots. Experimental results demonstrate a marked improvement in performance: on the custom dataset, the improved model achieves a stable mean average precision of approximately 92%. Specifically, ink spot detection reached a precision of 84.9% and recall of 77.7%, effectively reducing missed small-target defects; sintering defect detection attained 98.9% precision and 100% recall, addressing previous misclassifications due to background similarity; and scratch detection precision improved to 92.2%. Visual comparisons confirm that the enhanced model effectively overcomes the limitations of the original approach. By constructing a specialized dataset and implementing targeted, coordinated optimizations to the YOLOv8 architecture, this study significantly enhances the accuracy and robustness of screen-printing defect detection in photovoltaic cells, providing an effective solution for real-time online quality inspection in smart manufacturing lines.

## 1. Introduction

The quality of screen-printed grid lines on photovoltaic (PV) cells, as the core unit of PV modules, directly determines the module’s photoelectric conversion efficiency and long-term reliability. Micro-defects such as ink spots, line breaks, and scratches generated during the production process are key factors affecting product quality. Traditional machine vision inspection methods commonly encounter bottleneck issues when addressing those defects, including low detection efficiency, high rates of missed detection, and high false detection rates [1,2]. With the rapid development of deep learning technologies, neural network-based object detection algorithms have opened new pathways for the accurate detection of screen-printing defects in photovoltaic cells and achieved significant progress [3,4]. However, due to the characteristics of screen-printing defects—including their small size, dense features, low contrast with the background, and strong randomness in distribution—existing algorithms still face severe challenges in complex industrial environments. These challenges encompass insufficient detection accuracy for small targets, limited ability to distinguish between similar defects, and poor environmental adaptability [5,6,7]. To address these challenges, researchers worldwide have conducted extensive studies from various technical dimensions.

Although the aforementioned studies have made significant progress in defect detection accuracy, for the industrial real-time application scenario of photovoltaic cell printing defect detection, the algorithm inference speed, computational complexity, and hardware deployment costs represent equally critical determinants of practical feasibility. Currently, most published literature focuses on improving detection accuracy (mAP, recall), while discussions on the computational overhead (GFLOPs, parameter count) of models and their actual inference latency on industrial hardware remain insufficient. Chen [8] explored the combination of traditional image processing with deep learning. They employed improved grayscale integral projection, SVD difference methods, and a PSO-SVM classifier to handle specific defects, and optimized the approach using a YOLOv5 model enhanced with a CBAM attention mechanism. Nevertheless, issues of missed detection and misjudgment persist for micro-defects [9,10,11]. Wu et al. [12] developed a real-time compensation positioning system by integrating edge detection and line-fitting techniques, thereby improving detection efficiency. However, its template-matching mechanism exhibits limited adaptability to irregular defects. Kim [13] improved a ULBP-BP neural network model to refine recognition accuracy of texture defects, yet the high complexity of the model makes it difficult to meet the real-time requirements of high-speed production line, which typically demand millisecond-level response times. To meet the real-time and robustness requirements of industrial deployment, scholars have conducted in-depth exploration in preprocessing and noise suppression. Fu [14] proposed a dynamically weighted median filtering algorithm that effectively mitigated edge blurring caused by salt-and-pepper noise; however, its effectiveness in suppressing Gaussian noise and improving the signal-to-noise ratio remains limited. Li [15] and Smith [16] utilized wavelet transform-based multiscale analysis and adaptive frequency-domain filtering algorithms, respectively, to enhance the signal-to-noise ratio in specific scenarios. However, the former shows insufficient capability in capturing sub-pixel sharp features, while the latter faces stability challenges under dynamic lighting conditions. At the data level, Roanec [17] employed GANs for defect sample generation to alleviate the small-sample problem; however, discrepancies between generated data and real working conditions may lead to increase false positive rates. Significantly, none of these studies systematically quantify metrics such as frames per second (FPS) on typical industrial GPUs, inference time per image, or floating-point operations (FLOPs).

Recent research continues to advance performance boundaries. Wang [18] proposed and implemented the mechanical structure design, visual positioning algorithm, and automated control system for a solar cell screen-printing pipeline, optimizing the efficiency of PV cell electrode printing, though further improvements in detection accuracy are needed. Shanthi [19] achieved high-precision edge defect detection by combining image pyramids with contour analysis, but the issue of false detection still requires optimization. Li et al. [20] reconstructed YOLOv8 with multiple attention heads, significantly improving detection accuracy for micro-cracks, yet the added computational overhead introduced new challenges to real-time performance. Bin et al. [21] based on the YOLOv8 framework, significantly enhanced accuracy, efficiency, and generalization by introducing three strategies: the Vision-Aware Attention Convolution, the Transformer-Enhanced Task-Aligned Detection Head, and the Wise-IoU loss function. Although these YOLO-based variants perform excellently in general vision tasks, when directly applied to the specific scenario of photovoltaic cell screen printing defect detection reveals three key limitations: (1) insufficient small target perception, where downsampling loses fine-grained features, leading to poor localization accuracy and high missed detection rates; (2) efficiency–accuracy imbalance, as complex modules increase computational burden and inference latency, failing to meet real-time production requirements; (3) difficulty distinguishing similar defects, where standard classification heads struggle to capture subtle differences between sintering defects and scratches, resulting in confusion and high false detection rates [22].

In summary, although existing research has achieved significant results, photovoltaic screen-printing defect detection technology still faces some core bottlenecks in practical industrial applications include insufficient dynamic adaptability. Existing algorithms exhibit poor robustness to complex and variable factors such as lighting conditions, different cell–substrate materials, balance between precision and efficiency, and dust interference in production environments, which easily leads to missed or false detections; and the challenge of balancing accuracy and efficiency-under the high real-time requirements of industrial production lines, further improving detection accuracy algorithms for little and similar defects presents a significant challenge [23,24,25]. Therefore, this study aims to address these bottlenecks by constructing a high-quality dataset capable of simulating real working conditions and conducting collaborative optimization of the YOLOv8 algorithm model tailored for industrial scenarios. The goal is to significantly enhance the detection algorithm’s accuracy and robustness in complex environments while ensuring real-time performance, thereby providing a more effective online quality inspection solution for photovoltaic intelligent manufacturing.

## 2. Platform and Image Dataset

### 2.1. Construction of Image Acquisition Platform

Ensuring the clarity and quality of image features, the construction of a machine vision algorithm dataset is a crucial foundation for algorithm optimization. The quality of collected samples directly impacts the effectiveness of the entire algorithm training process. Traditional visual inspection methods, relying on single light sources and fixed depth-of-field acquisition systems, struggle to effectively suppress the impact of metal gridline reflections on image quality. Furthermore, issues like image noise and uneven illumination can significantly amplify measurement errors [26]. To address these challenges, this study designed a primary–secondary CCD multi-depth-of-field image acquisition platform. This platform enables the capture of multi-focal images of targets through the coordinated operation of multiple visual sensors. It primarily comprises visual and optical components, which require targeted selection based on the image characteristics of the object under inspection.

**Figure 1:**
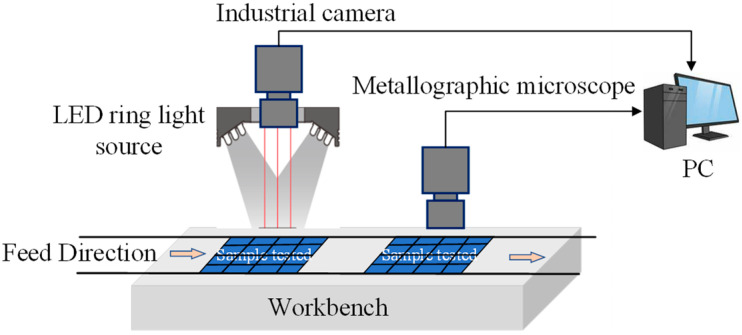
Schematic Diagram of the Primary–Secondary CCD Multi-Depth-of-Field Image Acquisition Platform Design.

Figure 1 illustrates the design principle of the primary–secondary CCD multi-depth-of-field image acquisition platform, employed for collecting image data on regarding defect morphology and microstructure of screen-printed photovoltaic surfaces. Based on the functional differences between the primary and secondary channels, visual components with different depths of field were selected, adopting a dual-channel cooperative imaging structure: The primary visual channel is equipped with an RS-A1300-GM&GC industrial camera (Kowa, Nagano City, Japan). With a working distance is set at 300 ± 5 mm, and a high frame rate of 300 fps meet the dynamic capture requirements of the motion platform. The secondary visual channel employs a MHAGO HanGuang BJ-A polarizing electron microscope (HanGuang Optics Co., Ltd., Wuxi, Jiangsu, China) system, equipped with a 5-megapixel imaging module, with an adjustable working distance range of 5–15 mm. When the light source illuminates the screen-printed sample surface, the visual components capture sample information, and the image data is subsequently stored in the system.

**Figure 2:**
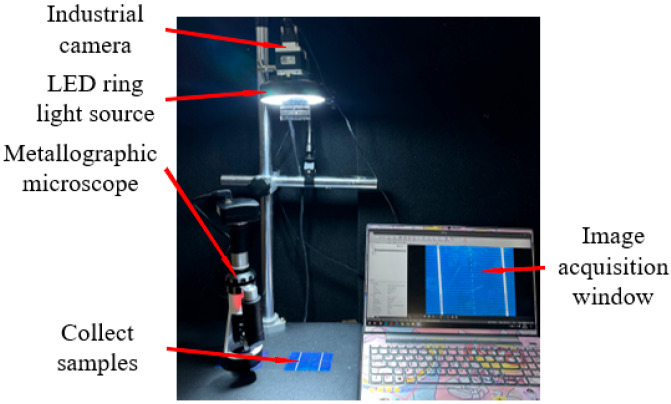
Main and auxiliary CCD multi-depth-of-field image acquisition platform.

Figure 2 shows the completed physical setup of the primary-secondary CCD multi-depth-of-field image acquisition platform. The selection of optical components is equally crucial for image data acquisition, especially considering the highly reflective nature of photovoltaic surfaces. Adjusting illumination intensity is essential to suppress reflections from metal gridlines [27]. Therefore, an LED ring light was selected for this platform, as it effectively separates target information from background interference. Its symmetrical optical path design helps eliminate shadows around the sample, accentuating features such as sample contours, scratches, and cracks. This maximizes the distinction between the measured information and background noise, demonstrating significant advantages in detecting surface scratches and defects.

### 2.2. Construction of Photovoltaic Screen Printing Image Dataset

Following the completion of the image acquisition platform, this study systematically compiled a dataset of photovoltaic screen-printing surface defect images for optimizing the YOLOv8 detection algorithm. The construction process involved four key stages: image acquisition, preprocessing, annotation, and partitioning, designed to cover diverse scenarios and varying conditions to enhance model generalizability.

**Figure 3:**
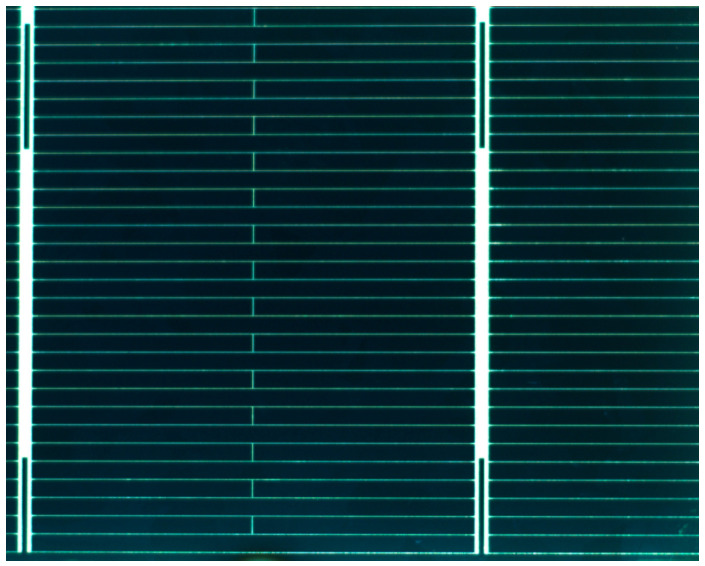
Sample of Photovoltaic Cell.

Figure 3 shows standard polycrystalline silicon photovoltaic cells (78 mm × 52 mm, 100 units) served as the base samples. Surface defects were categorized into three types: ink spots (0.5–1 mm), scratches (variable in size and shape), and sintering (areas ranging from ~10 mm × 40 mm to 10 mm × 60 mm) [28,29]. Each cell was rotated and repositioned, with an industrial camera capturing approximately 20 different views per sample, yielding around 2000 images. Among these, about 1940 contained valid defects, representing the targets under various poses and conditions. Given the limited defect categories and uniform background, this dataset is sufficient for training the YOLOv8 model [30].

**Figure 4:**
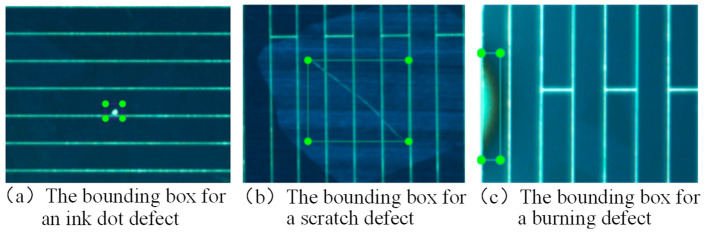
Rules for Classifying and Labeling Surface Defect Shapes.

The collected image data were then annotated. Following the labeling protocol of the YOLO series algorithms, each image required the generation of a corresponding annotation file that meets the specifications of the YOLOv8 algorithm. During the annotation process, bounding boxes were drawn individually around each defect, and corresponding class labels were assigned. Figure 4 illustrates the annotation rules for classifying different surface defect morphologies in photovoltaic cells: Figure 4a shows a bounding box identifying an ink spot defect, Figure 4b depicts a scratch defect, and Figure 4c represents a sintering defect. In the annotations, the labels “Ink dot,” “Burning,” and “Scratch” were used to identify ink spots, sintering, and scratches, respectively, and an annotation file was saved for each image. After completing all annotations, the dataset was scientifically partitioned: the training set was used for model learning and feature extraction, the validation set was used to monitor performance and adjust hyperparameters during training, and the test set was used for the final evaluation of the model’s generalization ability. The dataset was split into a 70%:30% ratio for the training validation set and test set, respectively. Within the 70% portion, 60% was allocated to the training set and 10% to the validation set, while the test set was fixed at 30%. This partitioning strategy effectively supports the transfer learning and performance validation of the YOLOv8 algorithm.

## 3. Methodology

### 3.1. YOLOv8 Algorithm Principles

YOLOv8 represents a typical deep learning-based real-time object detection algorithm, known for its high speed and ability to perform real-time detection in video streams. The algorithm employs a convolutional neural network (CNN) as its backbone, where multiple layers of convolutional and pooling operations automatically learn hierarchical semantic features—ranging from low-level to high-level semantic features, such as edges, textures, and colors—directly from images, providing robust feature representations for detection tasks [30].

**Figure 5:**
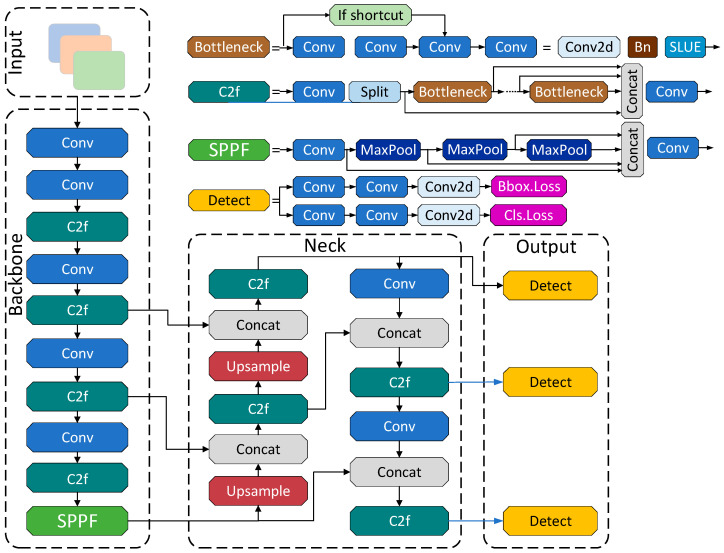
Structure diagram of the YOLOv8 network model.

Figure 5 shows the YOLOv8 network architecture primarily consists of three core components: the backbone, the neck, and the detection head. The backbone network serves as the feature extraction core of the model, drawing inspiration from the Cross-Stage Partial (CSP) Darknet structure to extract high-level semantic features that encompass target shape, texture, and contextual information from the input image. The neck network is responsible for fusing feature maps of different scales, enhancing the model’s ability to detect objects of varying sizes. Finally, the detection head performs target localization and classification based on the fused feature maps [31].

Specifically, the YOLOv8 backbone network incorporates the Cross-Stage Partial Network with Two Convolutional Layers and Feature Fusion (C2f) module and the Spatial Pyramid Pooling-Fast (SPPF) module. The C2f module integrates the lightweight design of YOLOv5’s CSP Bottleneck with 3 Convolutional Layers (C3) and the efficient aggregation mechanism of YOLOv7’s Efficient Layer Aggregation Network (ELAN). This architecture reduces computational overhead while mitigating gradient loss, thereby effectively improving detection accuracy [32]. The SPPF module enhances the model’s adaptability to multi-scale objects while maintaining a lightweight design by sequentially connecting multiple MaxPool layers.

In the neck section, YOLOv8 continues to employ the Feature Pyramid Network–Path Aggregation Network (FPN-PAN) structure, enabling efficient fusion of multi-scale features. The Head adopts a decoupled design that separates classification and regression tasks, and utilizes an anchor-free mechanism to simplify hyperparameter tuning and reduce model complexity. Regarding the loss functions, Binary Cross-Entropy (BCE) loss is applied for classification, while regression combines Distribution Focal Loss (DF Loss) with Complete Intersection over Union (CIOU) loss to enhance localization accuracy and training stability [33].

### 3.2. Optimization of the Main Network Structure

The backbone network, as the core component of an object detection model, is responsible for extracting multi-level features from input images. The effectiveness of its optimization directly determines the performance ceiling of subsequent feature fusion and target recognition. By improving convolutional layer structures and adjusting network depth and width, the model’s ability to capture semantic information of subtle defects on screen-printed photovoltaic surfaces—such as scratches and sintering—can be significantly enhanced, thereby providing more discriminative feature representations for the detection head.

To address the issues of low contrast between defects like scratches or sintering and dark backgrounds in screen-printed photovoltaic images, as well as the tendency for missed detection, the RepNCSPELAN4 module is introduced. This module combines cross-stage partial structures with an efficient layer aggregation mechanism to enhance multi-scale feature fusion and improve the model’s robustness in identifying subtle defects. Meanwhile, to better preserve the fine edges and texture information of scratch defects and avoid detail loss during traditional sampling, the WaveConv module is proposed, which embeds wavelet transform into convolutional operations to strengthen the extraction of high-frequency details. The following sections elaborate on and compare these two optimized structures in detail.

#### 3.2.1. RepNCSPELAN4 Module

**Figure 6:**
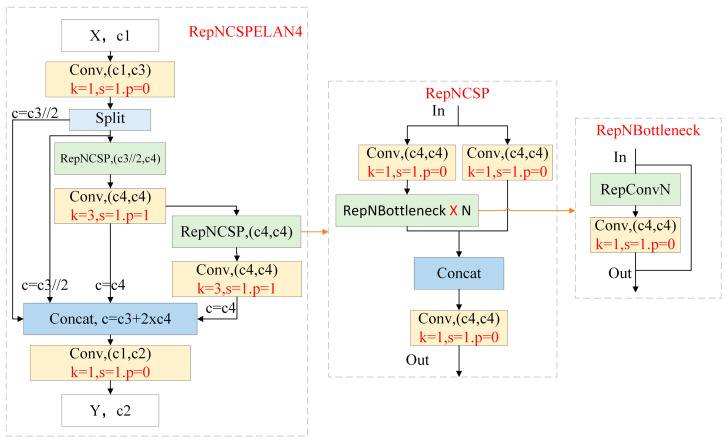
RepNCSPELAN4 algorithm network structure diagram.

The RepNCSPELAN4 module integrates the advantages of both the CSPNet and ELAN architectures: CSPNet reduces computational redundancy through gradient path planning, while ELAN provides an efficient multi-scale feature fusion strategy, thereby improving accuracy while maintaining high inference speed. Figure 6 shows the overall structure of RepNCSPELAN. Its core component, RepNCSP, consists of Conv and RepNBottleneck modules, where the RepNBottleneck module is specifically designed to enhance small-target features, thereby improving the detection capability for micro-defects. The input parameters of the module are [c1, c2, c3, c4], where c1 denotes the number of input channels (the output channel count of the preceding module), and c2 denotes the number of output channels. In the RepNCSP sub-module, the input channel count is c3, and the output channel count is c4. Configurable parameters for this sub-module include: repetition count n, whether to use skip connections (shortcut), the number of groups in group convolution g, and the expansion coefficient e that controls the internal channel expansion ratio. With reasonable parameter settings ([n = 1, shortcut = True, g = 1, e = 0.5]), this structure achieves an optimal balance between computational efficiency and detection accuracy while ensuring adequate feature representational capacity.

#### 3.2.2. WaveConv Module

To enhance the model’s perception of high-frequency detailed features and improve the efficiency of multi-scale feature extraction, this study constructs a WaveConv module based on wavelet transform. Designed to replace traditional downsampling operations in YOLOv8—such as strided convolution or max pooling—this module leverages the advantages of wavelet transform in time–frequency domain analysis. It achieves dimensionality reduction in feature maps while better preserving high-frequency details such as edges and textures, thereby effectively mitigating the detail loss typically caused by conventional pooling operations.

**Figure 7:**
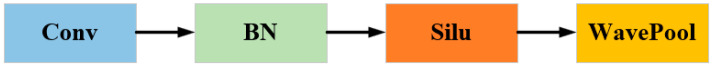
Wconv Module Network Structure Diagram.

The overall structure of the WaveConv module is illustrated in Figure 7. Its workflow consists of four core stages: first, standard convolution (Conv) extracts local features from the image; next, batch normalization (BN) standardizes the feature distribution to stabilize training and accelerate model convergence; subsequently, the Silu activation function is introduced to enhance the model’s nonlinear representational capacity; finally, the WavePool operation, based on wavelet transform, replaces traditional pooling to perform down sampling on the feature maps. Through the synergistic action of these operations, this structure forms a complete feature processing pipeline that balances detail preservation with computational efficiency [34].

**Figure 8:**
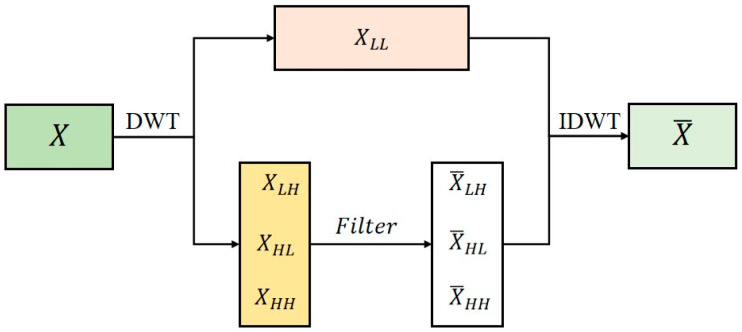
Network Structure Diagram of Wavelet Transform Process.

Figure 8 illustrates the core processing flow of the WavePool module. This module innovatively integrates wavelet transform into the pooling operation, achieving feature downsampling through a “decomposition–processing–reconstruction” mechanism. Its technical foundation is the wavelet transform theory. It first performs multi-scale decomposition of the input features, then enhances the sub-band features, and finally reconstructs the primary feature structures. This process effectively preserves detailed information while reducing the spatial resolution of the feature maps.

The Wavelet Transform, as a time-frequency domain analysis tool, can be implemented through the following steps The input is a feature map $X\epsilon R^{\left(B\times C\times H\times W\right)}$, where B is the batch size, C is the number of channels, and H and W are the height and width of the feature map, respectively. After processing by the Discrete Wavelet Transform (DWT), the output consists of four sub-band components: one low-frequency component $X_{LL}\epsilon R^{(B\times C\times \frac{H}{2}\times \frac{W}{2})}$, representing the overall contour information of the image. $X_{\mathrm{LH}}\epsilon R^{\left(B\times C\times \frac{H}{2}\times \frac{W}{2}\right)}$ (horizontal low-pass, vertical high-pass), capturing vertical edges, $X_{\mathrm{HL}}\epsilon R^{\left(B\times C\times \frac{H}{2}\times \frac{W}{2}\right)}$ (horizontal high-pass, vertical low-pass), capturing horizontal edges. $X_{HH}\epsilon R^{(B\times C\times \frac{H}{2}\times \frac{W}{2})}$ (horizontal high-pass, vertical high-pass), and capturing diagonal textures and details. Here, the low-frequency component $X_{LL}$ is obtained by applying a low-pass filter (L) in both the row and column directions. The high-frequency components $X_{LH}$, $X_{HL}$, and $X_{HH}$ are derived by introducing a high-pass filter (*H*) in the horizontal, vertical, and diagonal directions, respectively, to extract edge and texture features from different orientations. (2) $L_{0}$ and $H_{0}\epsilon R^{\left(\frac{H}{2}\times H\right)}$ be the low-pass and high-pass filter matrices for the row direction, $L_{1}$ and $H_{1}\epsilon R^{\left(\frac{W}{2}\times W\right)}$ be the filter matrices for the column direction. The input feature map X is decomposed by DWT into four sub-bands through the following computation:

(1) $$\left\{\begin{array}{c} X_{LL}=L_{0}\cdot X\cdot L_{1}^{⊤}\\ X_{LH}=H_{0}\cdot X\cdot L_{1}^{⊤}\\ X_{HL}=L_{0}\cdot X\cdot H_{1}^{⊤}\\ X_{HH}=H_{0}\cdot X\cdot H_{1}^{⊤} \end{array}\right.$$

This decomposition separates the image information into different frequency band components: contour (represented by ${\;X}_{LL}$), edges (represented by $X_{LH}$ and $X_{HL}$), and textures (represented by $X_{HH}$), laying the foundation for subsequent feature enhancement and reconstruction. (3) To enhance meaningful details and suppress noise interference, selective enhancement of the high-frequency sub-bands is necessary. This study employs dedicated high-frequency filters to process these components. Let the filtering operation function be Filter, applied to the three high-frequency components $X_{LH}$, $X_{HL}$, and $X_{HH}$, yielding the enhanced outputs:

(2) $$\left\{\begin{array}{c} {\bar{X}}_{LH}=Filter\left(X_{LH}\right)\\ {\bar{X}}_{HL}=Filter(X_{HL})\\ {\bar{X}}_{HH}=Filter(X_{HH}) \end{array}\right.$$

This filtering process can be designed in various forms-such as threshold filtering, enhancement convolution, or attention weighting-based on specific task requirements. Its goal is to strengthen high-frequency features (edges and textures) relevant to defects while suppressing irrelevant noise, thereby improving feature quality. (4) To restore the spatial structure of the image features, the processed frequency-domain sub-bands need to be reconstructed. This study employs the Inverse Discrete Wavelet Transform (IDWT) for feature reconstruction, calculated as follows:

(3) $$\bar{X}=I_{0}^{T}\cdot X_{LL}\cdot I_{1}+H_{0}^{T}\cdot {\bar{X}}_{LH}\cdot I_{1}+I_{0}^{T}\cdot {\bar{X}}_{HL}\cdot H_{1}+H_{0}^{T}\cdot {\bar{X}}_{HH}\cdot H_{1}$$

Here, $I_{0}$ and $I_{1}$ represent the reconstruction matrices for the row and column directions (typically related to the low-pass filter matrices from the decomposition stage), while $I_{0}$ and $I_{1}$ are the corresponding high-pass reconstruction matrices. During reconstruction, the low-frequency component $X_{LL}$ preserves the main structural information of the image. The filtered and enhanced high-frequency components ${\bar{X}}_{LH}$, ${\bar{X}}_{HL}$, and ${\bar{X}}_{HH}$ provide detailed features in the horizontal, vertical, and diagonal directions, respectively. By weighted fusion of all sub-band components, the final enhanced feature map $\bar{X}$ is output, significantly improving detail representation capability while retaining the global structure.

Let the input feature map be $X_{in}{\epsilon R}^{B\times C_{in}\times H\times W}$, The forward pass of the WaveConv module can be formulated as:

(4) $$\left\{\begin{array}{c} X_{conv}=W_{conv}\cdot X_{in}+b_{conv}\\ X_{bn}=B_{N}(X_{conv})\\ X_{\sigma }=SiLU(X_{bn})\\ X_{out}=WavePool(X_{\sigma }) \end{array}\right.$$

Among them, ***W****_conv_* and *b_conv_* are the weight and bias of the standard convolution layer; ***B****_N_* (·) represents the batch normalization operation; *SiLU*(·) is the Sigmoid Linear Unit activation function; *WavePool* (·) is a pooling operation based on the two-dimensional discrete wavelet transform (DWT), whose internal process follows Equations (1)–(3) in the document. The core steps are: applying DWT to the input $X_{\sigma }$ to obtain four subbands *X_LL_*, *X_LH_*, *X_HL_*, *X_HH_*; applying a learnable filtering function Filter(·) to the high-frequency subbands *X_LH_*, *X_HL_*, *X_HH_* to obtain enhanced subbands *X_LH_*, *X_HL_*, *X_HH_*; and reconstructing the downsampled feature map $X_{out}{\epsilon R}^{B\times C_{in}\times H/2\times W/2}$ from the low-frequency subband *X_LL_* and the enhanced high-frequency subbands via the inverse discrete wavelet transform (IDWT).

### 3.3. Optimization of the Neck Network Structure

**Figure 9:**
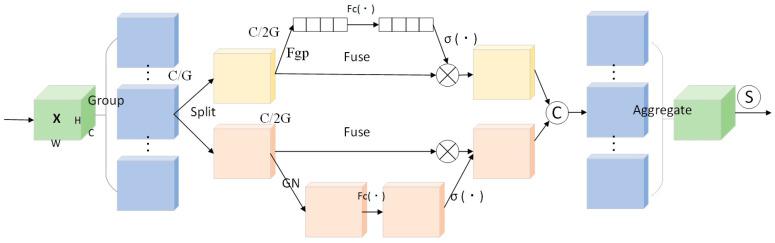
SA Mechanism Algorithm Network Structure Diagram.

The neck network plays a crucial role in multi-scale feature fusion within object detection models. Its optimization aims to enhance information interaction between features from different levels, thereby improving the model’s detection performance for multi-scale targets. However, while the mentioned improvements to the backbone network enhanced feature representation capability, they also introduced additional computational overhead. Therefore, optimizing the neck network requires balancing feature fusion effectiveness with model lightweighting. To this end, this study proposes two complementary optimization strategies. First, the Shuffle Attention (SA) module is introduced to cross-reorganize attention across channel and spatial dimensions, strengthening key feature representation while reducing model complexity. Second, an innovative Refined Detail Processing (RDP) module is constructed. This module integrates a detail-processing mechanism to specifically enhance target edges and high-frequency details and employs 1 × 1 convolutions for channel dimension compression to effectively control the total parameter count. The structure and mechanisms of these two modules are analyzed and compared in detail below: (1) the structure of the SA module is shown in Figure 9. Its core concept is to achieve synergistic enhancement across channel and spatial dimensions through feature grouping and attention weight reconstruction, thereby improving feature discriminability while maintaining relatively low computational complexity. This module first uses Shuffle Units to partition the channel dimension of the input feature map into multiple non-overlapping subgroups. Channel and spatial attention are computed separately within each subgroup for parallel feature processing. Subsequently, a Channel Shuffle operation facilitates information exchange between different subgroups, culminating in the aggregated output of all sub-features. Let the input feature map be $X\epsilon R^{C\times H\times W}$, where C, H, and W represent the number of channels, height, and width, respectively. First, X is uniformly partitioned along the channel dimension into G non-overlapping subgroups. The dimension of each subgroup $X_{k}$ is $R^{C/G\times H\times W}$, as shown in Equation (5):

(5) $$X=[X_{1},\ldots ,X_{G}],X_{k}\in R^{C/G\times H\times W}$$

Next, each subgroup $X_{k}$ is further split into two branches, $X_{k1}$ and $X_{k2}$, each with a dimension of $R^{C/(2G)\times H\times W}$, as shown in Equation (6):

(6) $$X_{k1},X_{k2}\in R^{C/(2G)\times H\times W}$$

Here, the $X_{k1}$ branch generates the channel attention map, while $X_{k2}$ generates the spatial attention map. In the channel attention branch, global average pooling is first applied to obtain global context information $s\in R^{C/2G\times 1\times 1}$, calculated as shown in Equation (7):

(7) $$s=\frac{1}{H\times W}\sum_{i=1}^{H}\sum_{j=1}^{W}X_{k1}(i,j)$$

Subsequently, a fully connected layer containing weights $W_{1}$ and bias $b_{1}$, followed by the Sigmoid activation function σ, is used to generate attention weights. These weights are then applied to the original features to produce the enhanced output $X_{k1}^{\prime }$, as shown in Equation (8):

(8) $$X_{k1}^{\prime }=\sigma (F_{c}(s))\times X_{k1}=\sigma (W_{1}s+b_{1})\times X_{k1}$$

In the spatial attention branch, Group Normalization (GN) is first applied to $X_{k2}$. Then, a convolutional layer (with weight $W_{2}$ and bias $b_{2}$) combined with the Sigmoid activation function generates spatial attention weights, which are multiplied with the original features to yield the output $X_{k2}^{\prime }$, as shown in Equation (9):

(9) $$X_{k2}^{\prime }=\sigma (W_{2}\times \mathrm{GN}(X_{k2})+b_{2})\times X_{k2}$$

Finally, the outputs from the two branches are concatenated. A Channel Shuffle operation enables cross-group information exchange, producing the final output for this subgroup, $X_{k}^{\prime }\in R^{C/G\times H\times W}$, as shown in Equation (10):

(10) $$X_{k}^{\prime }=[X_{k1}^{\prime },X_{k2}^{\prime }]\in R^{C/G\times H\times W}$$

2 RDP Module

**Figure 10:**
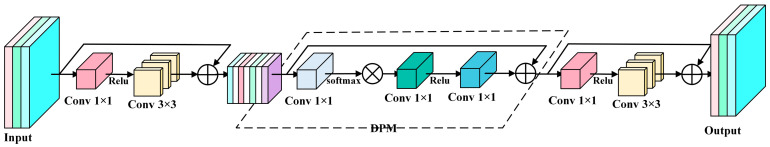
Network Structure Diagram of RDP Algorithm.

Figure 10 illustrated the structure of the Refined Detail Processing (RDP) module, which enhances detailed features while controlling model complexity, this module deeply integrates a detail-processing mechanism. Its core optimization strategies are twofold. First, it generates spatial weights via a convolutional mask (conv_mask) and the Softmax function to enhance target edges and high-frequency details within the feature map, effectively suppressing background noise interference. This is particularly suitable for features like scratches that are easily confused with the background. Second, it adopts a lightweight design, using 1 × 1 convolutions to replace complex operations in traditional attention mechanisms. This adjusts the number of channels without introducing excessive parameters, significantly improving computational efficiency [35].

As shown within the dashed box in Figure 10, the DPM (presumably a component of RDP) achieves detail enhancement through a spatial attention mechanism. The specific computational flow is as follows:

(1) Let the input feature map be X:

(11) $$X\;\in R^{B\times C\times H\times W}$$

where B: batch size (number of data samples per input); C: number of channels; H, W: height and width of the feature map.

(2) Generate the spatial attention weight mask M using a 1 × 1 convolution:

(12) $$M={\mathrm{Conv}}_{1\times 1}(X),M\in R^{B\times 1\times H\times W}$$

This convolutional layer contains no bias term, outputting a single-channel weight map.

(3) Apply Softmax normalization to M along the spatial dimensions (H×W) to obtain the attention weights A:

(13) $$A\;=\;Soft\max(M),\;A\in R^{B\times 1\times H\times W}$$

After normalization, the condition is satisfied:

(14) $$\sum_{i=1}^{H}\sum_{j=1}^{W}A_{b,1,i,j}=1$$

(4) Use A as weights to perform a weighted aggregation on the input feature map X, extracting the global contextual feature G:

(15) $$G=\;\sum_{i=1}^{H}\sum_{j=1}^{W}A_{b,1,i,j}\cdot X_{b,:,i,j},\;G\;\in R^{\left(B\times C\times 1\times 1\right)}$$

(5) Adjust the channel dimensions of G through two layers of 1 × 1 convolutions with ReLU activation:

(16) $$G^{\prime }=W_{2}\cdot \mathrm{ReLU}(W_{1}\cdot G),{\;G}^{\prime }\in R^{B\times C\times 1\times 1}$$

where $W_{1}\in R^{C\times C}$ and $W_{2}\in R^{C\times C}$ are the weight matrices of the 1 × 1 convolutions.

(6) Add the adjusted feature $G^{\prime }$ element-wise to the original input X to obtain the final output Y:

(17) $$Y=X+G^{\prime },\;Y\in R^{B\times U\times H\times W}$$

### 3.4. Improved YOLOv8 Model

**Figure 11:**
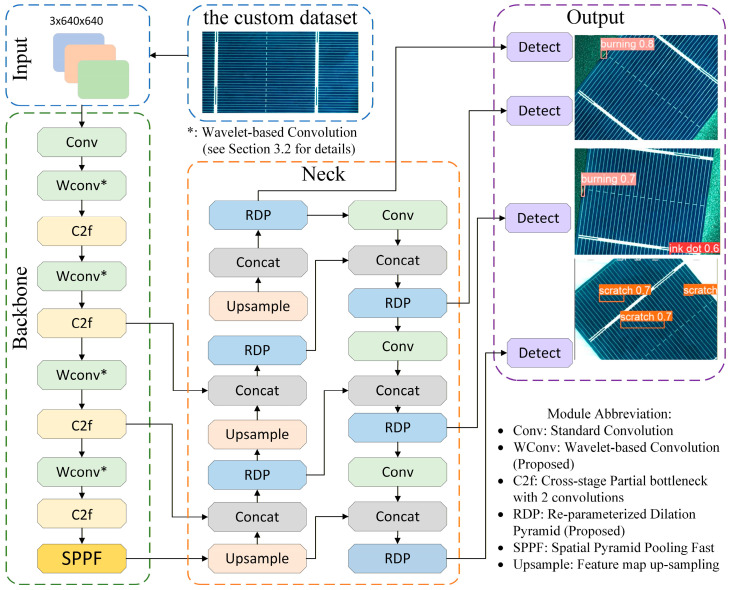
Architecture of the proposed YOLOv8 model integrated with the WaveConv and RDP modules.

As the number of downsampling layers in the network increases, the receptive field of the feature maps expands accordingly. While this facilitates the capture of global semantic information and enhances overall detection performance, an excessively large receptive field can render the model to become overly sensitive to medium and large targets while missing small defect targets. To resolve this trade-off, this study introduces a dedicated small-target detection head for detecting micron-level ink spot defects, based on the original three-head architecture of YOLOv8. The overall structure of the improved YOLOv8 model is shown in Figure 11. The main optimizations are reflected in the following three aspects: First, the reconstruction and enhancement of the small-target detection head improve the model’s perception of tiny ink spot defects, effectively enhancing the representation quality of small targets in deep features. Second, to address the difficulty of distinguishing sintering defects from the background due to their high color similarity, a WaveConv module based on wavelet transform is introduced. This module improves feature fusion by enhancing multi-scale feature extraction capability, thereby strengthening the recognition of low-contrast defects. Finally, to tackle the challenge posed by the variable morphology of scratch defects and their susceptibility to background noise interference, the RDP module is constructed. By enhancing edge and detail information in features, it effectively suppresses the influence of polycrystalline silicon background noise [36]. These improvement measures work synergistically to collectively enhance the model’s detection accuracy and robustness for multiple defect types in complex scenarios.

The following training configuration for improved YOLOv8 model. The SGD optimizer is used with an initial learning rate of 0.01, momentum of 0.937, and weight decay of 0.0005. Training lasts for 300 epochs with a batch size of 64. A linear warmup strategy is applied initially, followed by a cosine annealing schedule that reduces the learning rate to a final value of 0.0001. The data augmentation pipeline includes Mosaic (enabled for the first 90% of epochs), MixUp with probability 0.1, random horizontal flipping (*p* = 0.5), HSV enhancement (hue shift ±1.5%, saturation shift ±70%, value shift ±40%), and random affine transformation (degrees = 0.0, translate = 0.1, scale = 0.5, shear = 0.0). The total training duration is 120 s on a single NVIDIA V100 GPU.

## 4. Samples and Experiments

### 4.1. Evaluation Metrics

To comprehensively evaluate the performance of the algorithmic model, precision, recall, and mean average precision (*mAP*) were selected as the core evaluation metrics. These metrics provide a quantitative assessment of the model from three dimensions: precision capability, recall capability, and overall detection performance. Their computational relationships are as follows [37]:

(18) $$\left\{\begin{array}{c} P=\frac{TP}{TP+FP}\\ R=\frac{TP}{TP+FN}\\ mAP=\frac{1}{C}\int_{0}^{1}\sum_{c=1}^{C}P_{c}(R)dR \end{array}\right.$$

Here, True Positive (*TP*) represents the number of correctly identified positive samples, False Positive (*FP*) is the number of negative samples incorrectly identified as positive, False Negative (*FN*) is the number of positive samples that were missed, and *C* is the number of defect categories. The *mAP*, by computing the average precision across all categories at different recall rates, comprehensively reflects the model’s overall performance in both localization and classification tasks.

### 4.2. Experimental Comparison and Analysis

**Figure 12:**
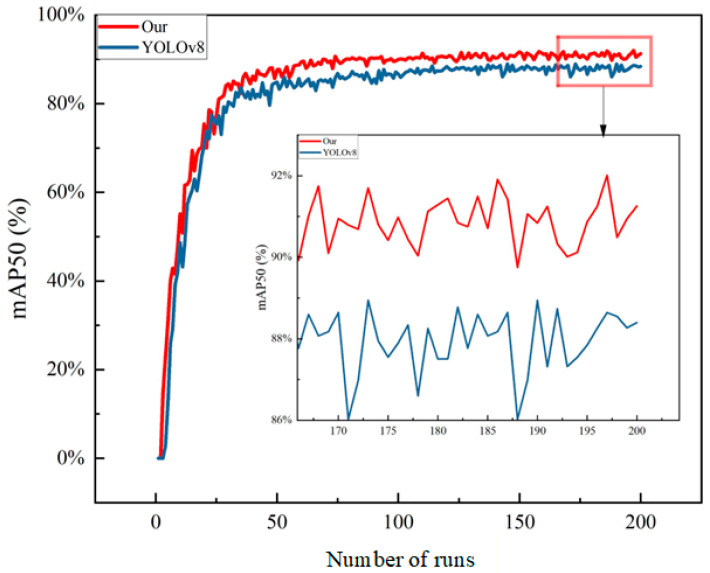
Line chart of mAP50 for the algorithms before and after optimization.

**Figure 13:**
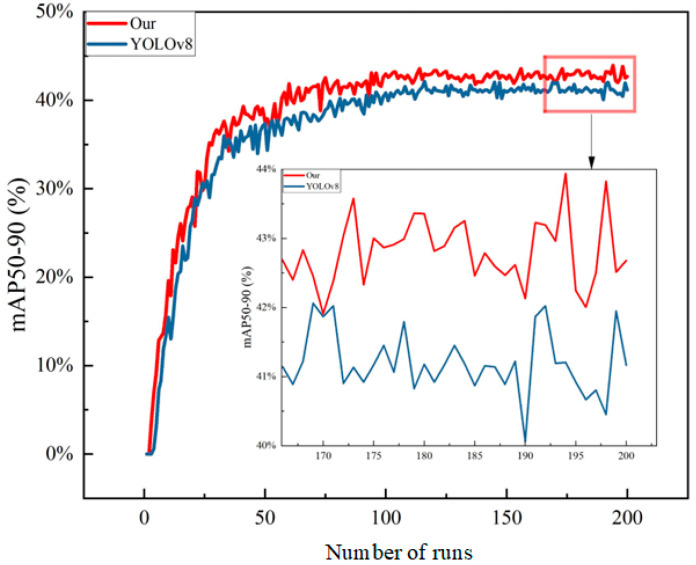
Line chart of mAP50-95 for the algorithms before and after optimization.

To verify the effectiveness of the proposed improvements, systematic comparative experiments were conducted on the YOLOv8 model before and after optimization. Figure 12 and Figure 13 show the performance comparison curves at an Intersection over Union (IoU) threshold of 0.5 (mAP@50) and across the interval of 0.5 to 0.95 (mAP@50-95), respectively. The mAP@50 primarily reflects the model’s detection capability under lower localization accuracy requirements, while mAP@50-95 provides a stricter evaluation of the model’s comprehensive performance under high localization accuracy demands. Figure 12 shows the mAP50 line chart. The blue curve represents the average detection precision of the optimized algorithm, which surpassed that of the original algorithm after approximately 50 training epochs and eventually stabilized around 92% without a declining trend, indicating effective identification of most targets.

Figure 13 shows the mAP50-95 line chart. It can be observed that the original algorithm exhibited a peak around 120 epochs; however, with further training, its performance showed a declining trend instead of improvement. In contrast, the detection precision of the optimized algorithm gradually increased with the number of training epochs.

The experimental results show that the mAP@50 of the optimized model surpassed that of the original model after about 50 training epochs and ultimately stabilized at approximately 92%, indicating strong rapid convergence capability and stability. Regarding the mAP@50-95 metric, the original model reached a peak around 120 epochs before its performance declined, whereas the optimized model’s performance continued to improve with increasing training epochs, demonstrating superior generalization capability and resistance to overfitting.

The loss function exhibited consistent convergence during the training process, with similar trends observed on both the training and validation sets. This indicates a stable training process without significant overfitting. All evaluation metrics (precision, recall, mAP) improved progressively during training and eventually stabilized, confirming that the improved YOLOv8 model achieved a high level of performance in target localization, classification, and overall detection.

Table 1 provides a detailed comparison of the performance for the three defect categories before and after optimization. It is evident that the optimized model shows significant improvement in the majority of metrics. Specifically, precision and recall for ink spot defects increased by 7.9% and 5.2%, respectively, indicating clear improvements in reducing both false positives and missed detections. The precision for sintering defects increased to 98.9%, with recall reaching 100%, signifying complete recall of this defect type and a significant enhancement in detection completeness. For scratch defects, precision increased by 0.9%, while recall slightly decreased by 0.8%. This reflects a typical precision-recall trade-off, where the model reduces false positives at the cost of a marginal increase in missed detections. Considering the substantial improvements in the other two defect categories and the overall increase in the average F1-score (or mAP), the optimized model demonstrates a net positive gain in comprehensive detection performance [38].

### 4.3. Ablation Studies

To validate the effectiveness and necessity of each proposed optimization module—namely, the small object detection head, Wave Conv module, and RDP module—in enhancing the performance of the YOLOv8 model, systematic ablation experiments were designed and conducted. By employing a controlled variable approach, optimization modules were progressively integrated to isolate and quantitatively evaluate the individual and combined contributions of each module to the model’s detection accuracy (mAP) and computational complexity (GFLOPs, parameter count).

All experiments were performed under identical training datasets, validation environments, and hyperparameter settings. A total of five comparative experiments were designed: Experiment 1 (Baseline): Original YOLOv8 model. Experiment 2: Baseline model with only the small object detection output layer incorporated. Experiment 3: Baseline model with only the Wave Conv module applied to the backbone network. Experiment 4: Baseline model incorporating both the small object detection layer and the Wave Conv module. Experiment 5 (Full Model): Baseline model integrating all improvements, including the small object detection layer, Wave Conv module, and RDP module. The experimental results are presented in Table 2.

### 4.4. Visualization of Detection Result Analysis

**Figure 14:**
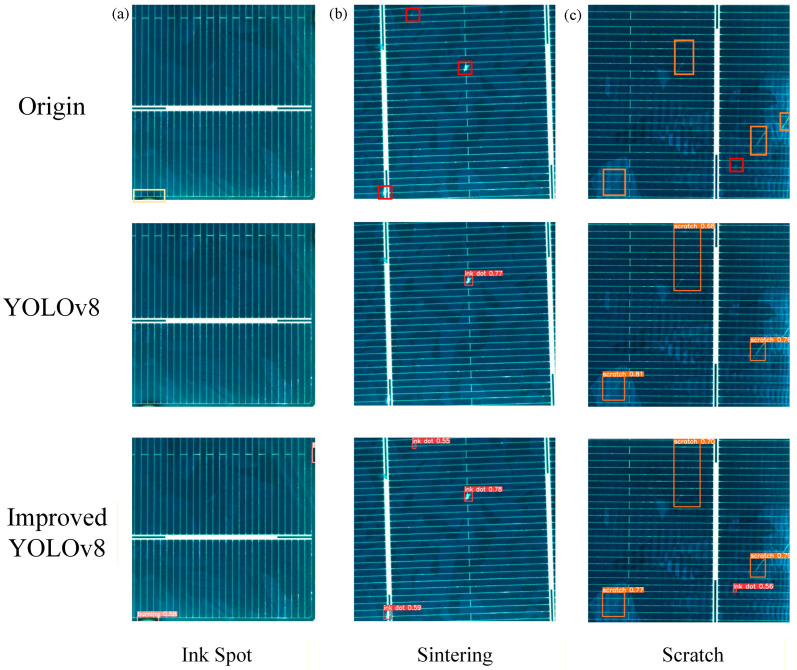
Comparison of actual detection effects between the original and optimized YOLOv8 algorithms.

To further validate the detection performance of the optimized model under practical conditions, a comparative experiment and theoretical analysis between the optimized YOLOv8 algorithm and the original model were conducted, with the results presented in Figure 14 [39]. Three sample images containing different defect types were randomly selected for visual comparison. Figure 14a shows the original YOLOv8 model failed to detect one sintering defect, mistakenly classifying the flawed region as normal. Additionally, it missed two ink spot defects in Figure 14b and one ink spot defect in Figure 14c, indicating its limited ability to identify small ink spots and sintering defects that closely resemble the background. In contrast, the optimized model successfully identified all three defect types across all samples without any missed or false detections. These visual outcomes intuitively confirm the effectiveness and robustness of the proposed enhancements in complex scenarios. The optimized model exhibits a marked improvement in detecting small targets and low-contrast defects [40].

## 5. Conclusions

To address the issues of missed detections, false positives, and difficulties in small target recognition in screen-printed photovoltaic surface defect detection, this study proposes an improved detection method based on an enhanced YOLOv8 model. Through systematic model architecture optimization and data enhancement strategies, the accuracy and robustness of defect detection have been significantly improved. The main conclusions of this research are as follows:

(1) To address the challenges posed by the highly reflective nature of photovoltaic cell surfaces and the small, complex morphology of defects, a multi-depth-of-field image acquisition platform based on coordinated primary and secondary CCDs was designed and constructed. By integrating a high-frame-rate industrial camera with a high-resolution electron microscope and employing a ring LED light source to effectively suppress reflections, this platform successfully established a high-quality dataset encompassing three types of defects: ink spots, scratches, and sintering. This provides substantial data support for model training.

(2) Improvements were made to YOLOv8 model, and the RepNCSPELAN4 module was introduced into the backbone network to enhance multi-scale feature fusion. Furthermore, an innovative Wave Conv module based on wavelet transform was designed, leveraging its sensitivity to edges and textures to better preserve detailed information of micro-defects during the pooling process. In the neck network, the integration of a lightweight Shuffle Attention mechanism and an innovative detail-processing module enhanced the expression of key features while effectively controlling model complexity. Additionally, a dedicated small-target detection head was added to specifically address micron-level ink spot defects. These synergistic improvements substantially strengthened the model’s capability in feature extraction and discrimination for defects resembling the background and for small targets.

(3) Experimental results on the custom-built dataset demonstrate that the optimized model’s overall detection performance is significantly superior to that of the original YOLOv8 model, with the mAP metric stabilizing at approximately 92%. Specifically, for the most challenging ink spot defects, precision and recall were improved to 84.9% and 77.7%, respectively. Recall for sintering defects reached 100%. The precision for scratch defects was increased to 92.2%. Despite optimized model effectively overcomes the original model’s shortcomings, such as missed detections of small targets and misjudgments of similar defects, this study has several limitations. First, the dataset lacks diversity, as it was collected from a single production line, limiting generalization to varied industrial environments. Second, deployment optimization for edge devices model quantization or TensorRT acceleration was not explored. Third, extremely small defects still suffer from non-negligible missed detections. Future work will address these issues by expanding dataset diversity using generative models, applying knowledge distillation and pruning for lightweight deployment, and exploring transformer-based architectures to further improve microdefect detection accuracy.

## Figures and Tables

**Table 1:** Comparison of detection results for surface defect categories before and after algorithm optimization.

Category	YOLOv8		Improved YOLOv8	
	Precision P/%	Recall R/%	Precision P/%	Recall R/%
Ink Spot	77	72.5	84.9	77.7
Sintering	97.3	98.7	98.9	100
Scratch	91.3	90.1	92.2	89.3

**Table 2:** The results of ablation studies.

	Model Configuration	GFLOPs/G	Number of Parameters	mAP/%	Rising Rate
1	YOLOv8	8.1	3,006,233	88.2	—
2	YOLOv8+ small target	12.5	2,977,852	89.7	+1.5%
3	YOLOv8+Wave Conv	11.0	3,006,713	89.7	+1.5%
4	YOLOv8+small target+Wave Conv	15.4	2,978,332	91.7	+3.5%
5	Improved YOLOv8	15.5	3,054,924	92.0	+3.8%

## Data Availability

The original contributions presented in this study are included in the article. Further inquiries can be directed to the corresponding authors.
